# Binary Graft of Poly(*N*-vinylcaprolactam) and Poly(acrylic acid) onto Chitosan Hydrogels Using Ionizing Radiation for the Retention and Controlled Release of Therapeutic Compounds

**DOI:** 10.3390/polym13162641

**Published:** 2021-08-09

**Authors:** Alejandra Ortega, Abigail Sánchez, Guillermina Burillo

**Affiliations:** Departamento de Química de Radiaciones y Radioquímica, Instituto de Ciencias Nucleares, Universidad Nacional Autónoma de México, Circuito Exterior, Ciudad Universitaria, Ciudad de México 04510, Mexico; alejandra.ortega@nucleares.unam.mx (A.O.); abisan03@live.com.mx (A.S.)

**Keywords:** radiation grafting, chitosan crosslinked, diclofenac release

## Abstract

In this study, we carried out the synthesis of a thermo- and pH-sensitive binary graft, based on *N*-vinylcaprolactam (NVCL) and pH sensitive acrylic acid (AAc) monomers, onto chitosan gels (*net*-CS) by ionizing radiation. Pre-oxidative irradiation and direct methods were examined, and materials obtained were characterized by FTIR-ATR, thermogravimetric analysis (TGA), scanning electron microscopy (SEM), and swelling tests (equilibrium swelling time, critical pH, and temperature). The best synthesis radiation method was the direct method, which resulted in the maximum grafting percentages (~40%) at low doses (10–12 kGy). The main goal of this study was the comparison of the swelling behavior and physicochemical properties of *net*-CS with those of the binary system *(net*-CS)-*g*-NVCL/AAc with the optimum grafting percentage (~30%). This produced a material that showed an upper critical solution temperature (UCST) of 33.5 °C and a critical pH value of 3.8, indicating the system is more hydrophilic at higher temperatures and low pH values. Load and release studies were carried out using diclofenac. The grafted system (32%) was able to load 19.3 mg g^−1^ of diclofenac and release about 95% within 200 min, in comparison to *net*-CS, which only released 80% during the same period. When the grafted system was protonated before diclofenac loading, it loaded 27.6 mg g^−1^. However, the drug was strongly retained in the material by electrostatic interactions and only released about 20%.

## 1. Introduction

**Figure 1:**
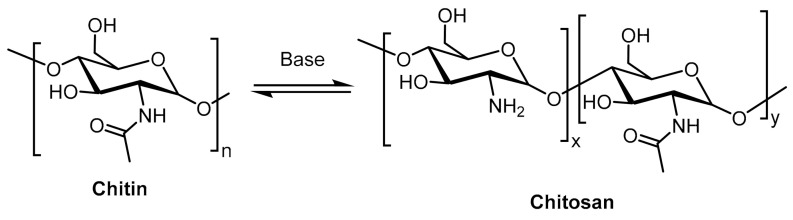
Partial deacetylation of chitin to produce chitosan.

Chitosan (CS) is a linear polysaccharide composed of random structural units of D-glucosamine and N-acetyl-D-glucosamine, and obtained through the partial deacetylation of chitin (Figure 1). CS is an important material in several applications because of its inherent characteristics, such as biodegradability, nontoxicity, biocompatibility, and antimicrobial and antifungal activities [1,2,3,4,5]. 

Another important form is crosslinked chitosan (*net*-CS), which may be produced using a variety of methods, including radical polymerization and crosslinking reactions of polymer’s amine groups with dialdehydes such as formaldehyde or glutaraldehyde [6,7]. The latter method tends to be preferred because it does not cause degradation of the polymer, while still producing robust crosslinked networks. Moreover, *net*-CS eliminates the solubility of this natural polymer in acid media, allowing it to produce more chemically resistant and versatile substrates [8,9].

Although both CS and *net*-CS are generally advantageous due to their ability to retain metal ions and drugs, they nonetheless present inconveniences that prevent further applications. For instance, these materials are difficult to process, they have poor mechanical properties after swelling, and they are difficult to chemically modify because they are not very intrinsically reactive. Considering this, a viable option to improve their properties is chemical modification [10,11,12] by grafting of functional polymers onto the polysaccharide structure using ionizing radiation [13,14]. Although this may cause both degradation of the polymer and promote the formation of carboxyl groups on the material [15,16], radiation has proven to be an effective pathway to graft other compounds onto CS. For instance, grafting of polystyrene, polyacrylamide, polyacrylic acid, and poly(2-hydroxiethyl methacrylate), and binary grafts of poly(*N*-vinylcaprolactam) and poly(*N*, *N*-dimethylacrylamide) have all successfully been produced using γ-radiation-induced polymerization [17,18,19,20,21].

Therefore, the current project focused on the grafting of poly(*N*-vinylcaprolactam) and poly(acrylic acid) onto *net*-CS using gamma radiation, for their use in drug loading and the release of substrates, because the grafted polymers exhibit thermal responsiveness and pH sensitivity, respectively. When they are combined, both properties may work synergistically to provide better loading and release of therapeutic agents. In this work, radiation doses were varied in the interest of finding optimal conditions to produce the grafted *net*-CS under reproducible conditions. Furthermore, the synthesized systems were characterized by FTIR-ATR, TGA, and SEM. Swelling tests were used to determine the critical pH and solution temperature. In addition, preliminary studies on drug loading and release were carried out using diclofenac as a drug model.

## 2. Materials and Methods

Chitosan powder (Mw: 1.46 × 10^5^ and deacetylation degree 79%) [7], diclofenac, formaldehyde, and glacial acetic acid were purchased from Sigma Aldrich (Estado de México, México) and used as received. *N*-vinylcaprolactam (NVCL) and acrylic acid (AAc) were also purchased from Sigma Aldrich (Estado de México, México) and vacuum distilled prior to use. Gamma-ray irradiation was performed using a Gammabeam 651PT (Nordion International Inc., Ottawa, ON, Canada) (^60^Co gamma-ray source) with an average dose rate of 10 kGy h^−1^ with an activity of 63,200 Ci at the Institute of Nuclear Sciences of Universidad Nacional Autónoma de México (UNAM). The total attenuated reflectance Fourier transform infrared (ATR-FTIR) spectra of the different samples were recorded with a Perkin-Elmer spectrum 100 spectrometer (Perkin-Elmer Cetus Instruments, Norwalk, CT, USA) with a Universal ATR with a diamond tip, performing 16 scans. TGA analysis was performed on a TA Instruments TGA Q50 thermogravimetric analyzer (New Castle, DE, USA) at a heating rate of 10 °C min^−1^. pH measurements were measured using a HANNA HI4212 potentiometer and a HANNA HI 1331B combined glass electrode (HANNA Instrument, CDMX, México) calibrated through a three point calibration using Fisher Scientific reference pH buffers (pH = 4.0, 7.0, 10.0). SEM analyses were determined with a Tescan Mira 3 LMU field-emission scanning electron microscope (Tescan USA Inc., Warrendale, PA, USA). Dried samples were coated with Au under vacuum for 10 s using an SPI Module Sputter Coat.

### 2.1. Crosslinking of CS

Chitosan was crosslinked with formaldehyde by a Schiff base mechanism [4]. Formaldehyde (10% *v*/*v*) was added to an aqueous 3% *w*/*v* solution of CS containing acetic acid (5% *v*/*v*). The resulting solution was stirred for 10 min, then covered and left for 24 h at room temperature. Finally, it was dried by evaporation in a fume hood for 12 h. The crosslinked CS was extracted to eliminate the remaining uncrosslinked CS. For this, *net*-CS was magnetically stirred with acetic acid 1% (*v*/*v*) for 3 days, during which the solvent was changed every 12 h, and then stirred with distilled water until reaching a neutral pH, during which the medium was changed every 4 h. The resulting *net*-CS was dried at room temperature under vacuum. The crosslinking percentage (Gel %) was calculated with Equation (1), where W_l_ is the mass of the insoluble *net*-CS and W_0_ is the initial mass of CS.

(1) $$\mathrm{Gel}\;\left(\%\right)=\left(\frac{W_{1}}{W_{0}}\right)\times 100$$

### 2.2. Grafting of NVCL/AAc onto net-CS

Grafting of NVCL and AAc was carried out by the one-step procedure. Both direct (simultaneous) and oxidative pre-irradiation methods were used for the binary grafting synthesis [22]. In the direct method, *net*-CS and the two monomers in methanol solutions were irradiated simultaneously in the absence of oxygen to produce radicals on all species and the grafting reaction occurred on the *net*-CS reactive sites. In the oxidative pre-irradiation procedure, *net*-CS was irradiated in the presence of oxygen to form reactive peroxides and hydroperoxides on the substrate, which later were broken with heating to form radicals in the presence of monomers to produce the grafting. 

#### 2.2.1. Direct Method

Approximatively 200 mg of *net*-CS was placed inside borosilicate glass ampoules with 7 mL of a methanol solution of NVCL (15% *v*/*v*) and AAc (5% *v*/*v*), and left to rest for 48 h at room temperature. To avoid the evaporation of the methanol, the ampoules were sealed with caps and parafilm. The conditions chosen in this step were selected according to previously reported protocols with NVCL and DMAAM [23,24]. The solution was removed and then the ampoules with swollen *net*-CS were bubbled with Ar for 20 min to eliminate oxygen from the medium. Finally, the ampoules were sealed with heat. The samples were irradiated at a dose rate of 10 kGy h^−1^ with doses varying between 4 and 12 kGy. The irradiated samples were washed by stirring them with distilled water for 48 h at room temperature, during which the solvent was changed every 8 h, and then with methanol for 24 h to eliminate residual monomers and ungrafted homopolymer. Finally, samples were vacuum dried until reaching a constant weight. The grafting yield (Graft %) was calculated with Equation (2), where W_g_ is the mass after grafting and W_i_ is the initial weight of *net*-CS.

(2) $$\mathrm{Graft}\;\left(\%\right)=\left(\frac{W_{g}-W_{i}}{W_{i}}\right)\times 100$$

#### 2.2.2. Oxidative Pre-Irradiation Method

Approximatively 200 mg of *net*-CS was placed in a beaker and directly irradiated in the presence of air with doses of 2.5 to 15 kGy at a dose rate of 10 kGy h^−1^. Later, the irradiated samples were placed inside borosilicate glass ampoules with 7 mL of a methanol solution of NVCL (15% *v*/*v*) and AAc (5% *v*/*v*), and left resting for 48 h at room temperature. The solution inside the ampoules was then bubbled with Ar for 20 min to eliminate oxygen from the medium, and the ampoules were then sealed with heat. The ampoules were then heated at 60 °C for 6 h. Then, the samples were treated according to the procedure reported for the direct method. The grafting yield (Graft %) was calculated with Equation (2).

### 2.3. Characterization

#### 2.3.1. Equilibrium Swelling Time

The swelling of the different samples was determined by the gravimetric method. After immersing them in distilled water for different periods of time, the samples were weighed and immersed again until a constant weight was obtained. The swelling ratio was determined with Equation (3), where W_s_ and W_d_ are the mass of the swollen and dried samples, respectively.

(3) $$\mathrm{Swelling}\;\left(\%\right)=\left(\frac{W_{s}-W_{d}}{W_{d}}\right)\times 100$$

The equilibrium swelling time was established as the point in time at which the samples reached constant swelling percentages. 

#### 2.3.2. Critical Points (Temperature and pH)

The temperature critical point was determined by immersing the samples in distilled water at different temperatures and by measuring the swelling percentage of the samples at the equilibrium swelling time. The critical point was defined as the inflection point in the plot comparing swelling (%) and temperature. Similarly, the critical pH of the samples was determined by immersing the samples in buffer solutions at different pH values ranging from 2 to 9, and determining their swelling behavior at the equilibrium swelling time. The critical pH was defined as the inflection point in a plot comparing swelling (%) and pH.

#### 2.3.3. Drug Loading and Release

Samples (pristine or grafted *net*-CS) were swollen in water or buffer solution (pH: 5) for 24 h and frozen and lyophilized in order to prepare them for drug loading, which was performed by immersing the lyophilized sample in 10 mL aqueous solution of diclofenac (0.029 mg mL^−1^). The samples were placed under constant stirring at 40 °C. The loading of the drugs was determined by measuring the absorbance (at λ = 265 nm) of the aqueous solution surrounding the sample at different time intervals. 

For drug release determinations, the drug-loaded samples were removed from the drug solutions and dried for 24 h in vacuum, protecting them from light. Then, the samples were placed in 10 mL of buffer solution (pH: 7.4) and then placed under constant stirring at 37 °C. The release was determined by measuring the absorbance (at λ = 275 nm) by aliquots of buffer solution at different intervals of time.

## 3. Results and Discussion

### 3.1. Crosslinking of CS

**Figure 2:**
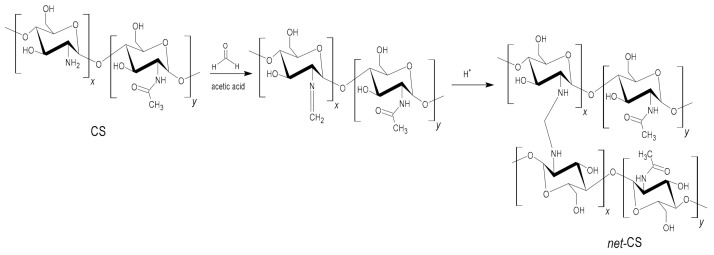
Crosslinking reaction of chitosan.

The gel percentage indicated an efficiency of crosslinking of 89%. This result is similar to others reported in the literature [4,17], indicating that CS was successfully converted to *net*-CS using 10% *v*/*v* formaldehyde solution (Figure 2). 

### 3.2. Effect of Dose on Grafting of NVCL/AAc onto net-CS

**Figure 3:**
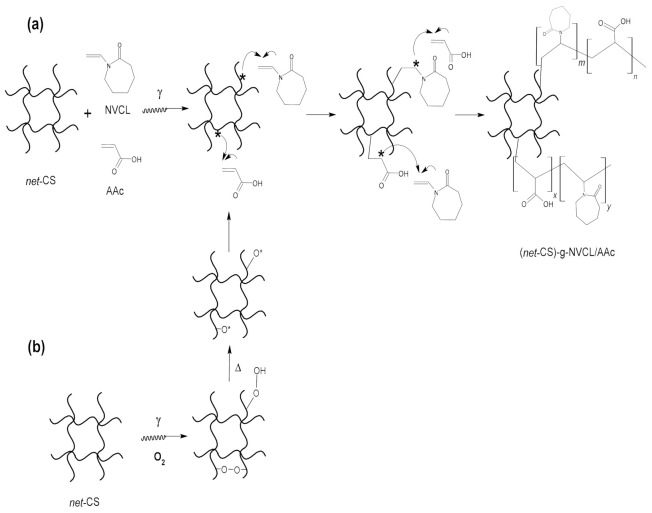
(**a**) Direct method and (**b**) oxidative pre-irradiation method for radiation grafting polymerization.

Binary grafting was carried out using direct and pre-iradiation methods because these methods vary greatly from each other due to the difference in the reagents’ interaction when radiation is directly applied to the monomers (Figure 3). Therefore, the grafting percentages obtained for both methods were compared.

**Figure 4:**
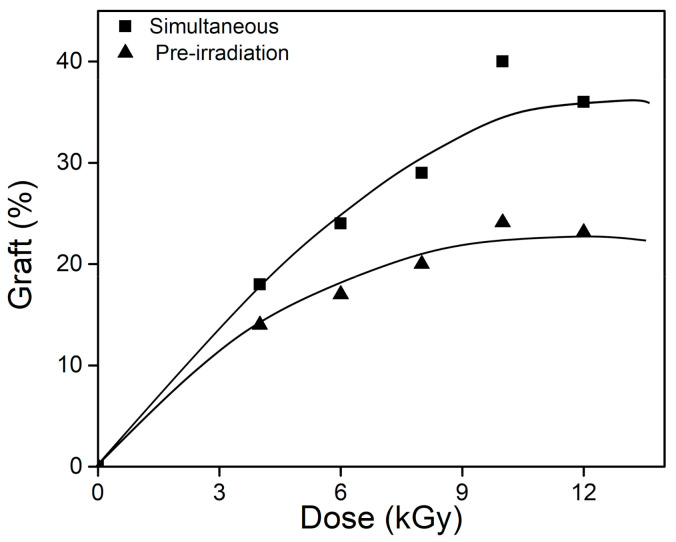
Binary graft percentage as a function of dose for ■ direct and ▲ pre-irradiation methods.

Simultaneous (direct) and oxidative pre-irradiation methods showed that the grafting percentage was directly proportional to the absorbed radiation dose, increasing steadily to a limit grafting value (Figure 4). Even when the behavior was similar, the direct method grafting yields were substantially higher, reaching grafting percentages up to 40%. In contrast, oxidative pre-irradiation only yielded grafting percentages of 24%. This difference may be because peroxide groups ruptured before the reaction with the monomers was achieved (macroradical recombination), and *net*-CS suffers a higher degradation when it is irradiated in the presence of air [25].

Although with these methods it is also possible to obtain an interpenetrated system rather than the graft copolymer, the latter process predominates because the main effect of radiation in CS is chain scission, which occurs via the breaking of glycosidic units, thus producing macroradicals in the CS structure [16]. Then, the recombination between the CS macroradicals and vinyl monomers starts the graft copolymerization [22]. Moreover, interpenetrating networks are favored when one crosslinked polymer is in the presence of another monomer/polymer that is more reactive to radiation [26], or when the polymers use different initiators to carry out the polymerization/crosslinking reaction [27]. The above results show that radiation was able to synthesize the binary graft *net*-CS-*g*-(NVCL/AAc), for which the direct method was the best option because it required less time and used lower doses at 10 kGy.

By comparsion, several authors have found that the optimum grafting percentages in polymeric systems for the retention and release of drugs were lower than 40% [28,29,30], because the length and entanglement of the grafted chains blocked drug diffusion, thus slowing the release. In addition, chitosan undergoes degradation when it is subjected to radiation, and this process is favored in the presence of oxygen. It is also necessary to mention that lower percentages present problems in terms of the apparent mechanical properties because the samples were broken when they swelled. 

This characterization was carried out using samples with grafts of 32% because our method allowed a reproducible grafting to be obtained and ensured that the CS degradation was low. 

### 3.3. Characterization

#### 3.3.1. FTIR

**Figure 5:**
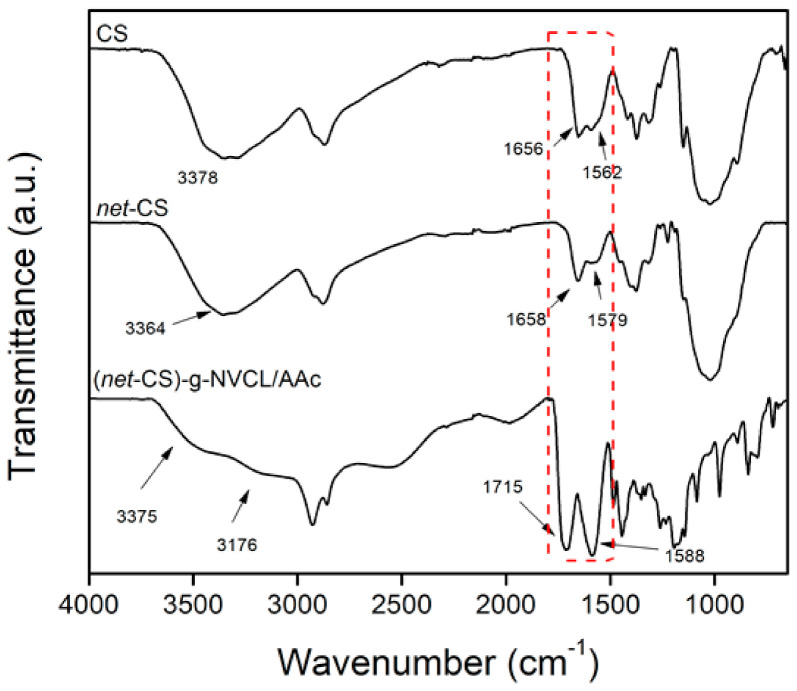
FTIR-ATR spectra of CS, *net*-CS, and (*net*-CS)-*g*-NVCL/AAc (32 % graft).

In the FTIR spectra (Figure 5), the signals corresponding to CS, *net*-CS, and (*net*-CS)-*g*-NVCL/AAc were compared to confirm the binary grafting. Firstly, in the CS spectrum, the characteristic peaks were found at 3378 cm^−1^ (O–H and N–H stretching), 2926 cm^−1^ (CH_2_ stretching of the pyranose ring), 1658 cm^−1^ (C=O stretching) corresponding to the remaining acetamide group, and 1562 cm^−1^ (N–H bending) [31]. When the crosslinking was carried out, *net*-CS showed the same peaks but the intensity of C=O stretching and N-H bending changed because the chitosan crosslinking mechanism was carried out via Schiff’s base mechanism, in which imine groups were formed [6]. However, these groups reacted with the other amine groups of chitosan and crosslinking took place. However, not all of the imine groups reacted due to steric hindrance, and some remained unreacted, which appeared in the IR spectrum overlapped with the carbonyl of CS at 1658 cm^−1^, whereas the signal of N–H bending decreased (1579 cm^−1^) due to the crosslinking reaction [32,33]. On the spectrum for (*net*-CS)-*g*-NVCL/AAc, in addition to the characteristic signals of *ne*t-CS, one additional peak at 1715 cm^−1^ was observed, which corresponds to the C=O stretching vibrations of both of the grafted monomers (carboxylic acid and amide groups). Another effect was the broadening of the peak at 3375 cm^−1^ due to the presence of OH in the carboxylic groups and the formation of hydrogen bonds between the carboxyl and hydroxyl groups [34]. Similarly, the peak at 1588 cm^−1^ was broader due to the amide groups of NVCL.

#### 3.3.2. Equilibrium Swelling

**Figure 6:**
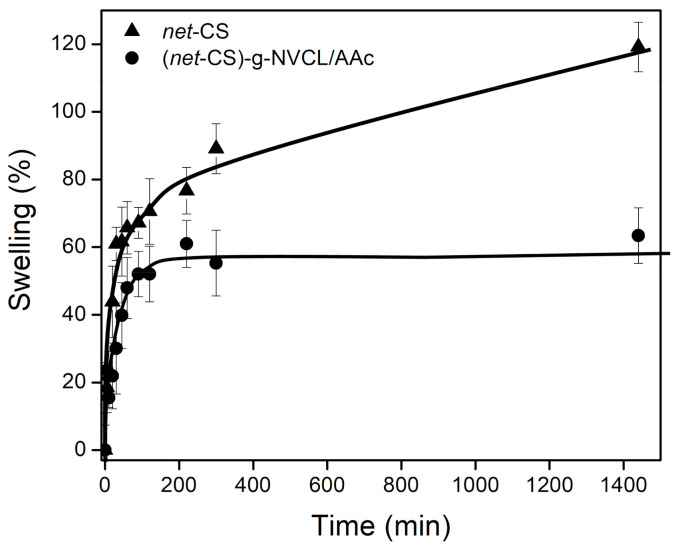
Equilibrium swelling in water of (▲) *net*-CS and (●) (*net*-CS)-*g*-NVCL/AAc (32% graft) at room temperature.

Equilibrium swelling (maximum swelling percentage) of pristine *net*-CS and the binary graft at 32% on *net*-CS were determined in distilled water at room temperature (Figure 6). The equilibrium swelling time for the pristine system was around 1400 min, whereas the binary grafted system reached the maximum swelling in 200 min. This could be because the maximum swelling percentage for the grafted system was lower than that of the pristine *net*-CS (~60% vs. ~120% respectively). The grafted *net*-CS absorbed less water because the grafted chains filled the empty voids of the CS networks. In addition, these chains contained NVCL, which was less hydrophilic than the D-glucosamine and N-acetyl-D-glucosamine monomeric units of chitosan. Moreover, at neutral pH, the grafted units of AAc (pKa = 4.2) onto *net*-CS were deprotonated (COO^−^), whereas amine groups of chitosan were protonated (NH_3_^+^), leading to the formation of electrostatic interactions that prevented the effective intake of water from the media. 

#### 3.3.3. SEM

**Figure 7:**
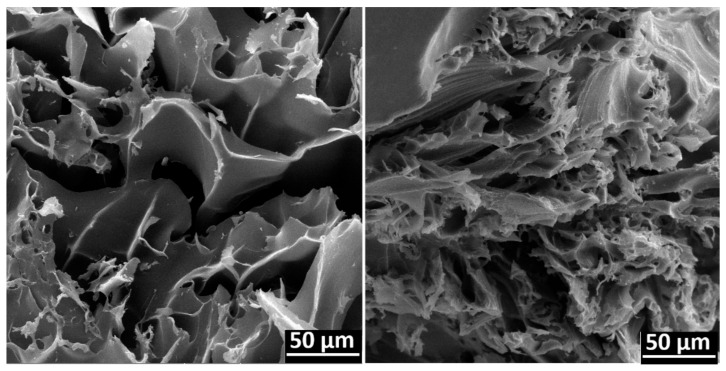
SEM micrographs of *net*-CS (**left**) and (*net*-CS)-*g*-NVCL/AAc 32% grafts (**right**).

SEM micrographs supported the graft reaction because they showed the different morphologies (Figure 7). Pristine *net*-CS showed a smoother structure than the grafted sample, which also presented thicker walls and a larger pore size, because the grafted chains filled pores and generated thin layers over the chitosan. Moreover, the grafted system presented a more compact structure but with a higher superficial area due to the grafted layers, which can improve the capacity to adsorb drugs. 

#### 3.3.4. Thermogravimetric Analysis

**Figure 8:**
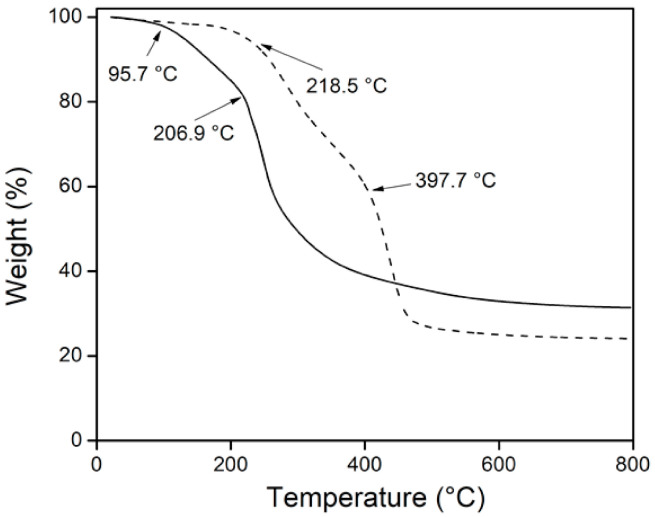
TGA results for *net*-CS (―) and (*net*-CS)-*g*-NVCL/AAc (32 %) (– –).

The grafting was also supported by TGA (Figure 8). *net*-CS showed a weight loss in two stages; the first was observed below at 100 °C due to the loss of moisture, perhaps bound water, which was eliminated when the sample was heated (4%). Furthermore, the second stage of decomposition was observed at 206.9 °C due to the depolymerization of chains by the breaking of glycoside units, during which there was a weight loss of 44.1%. The char residue was about 31.4% at 800 °C, which is in accordance with the literature [34]. In the binary grafting system, thermal decomposition was also carried out in two stages. The first started at 218.5 °C with a weight loss of 35%; this step was associated with chitosan decomposition. Thermal resistance increased due to electrostatic interactions between NH_2_ groups of CS and COOH of AAc. In addition, chitosan was degraded by gamma radiation, partially breaking the glycoside units while the monomers were grafting. This means that there was a lower weight percentage of glycosidic bonds that were thermally broken. The second stage around 400 °C corresponds to the decomposition of grafted chains because the moieties of caprolactam and carboxylic acid are thermally more stable than those of chitosan. The weight loss during this stage was 33.4%. Above 420 °C, the binary graft had a higher weight loss than chitosan, and obtained a char residue of 24.1% at 800 °C.

#### 3.3.5. Critical Temperature Determination 

**Figure 9:**
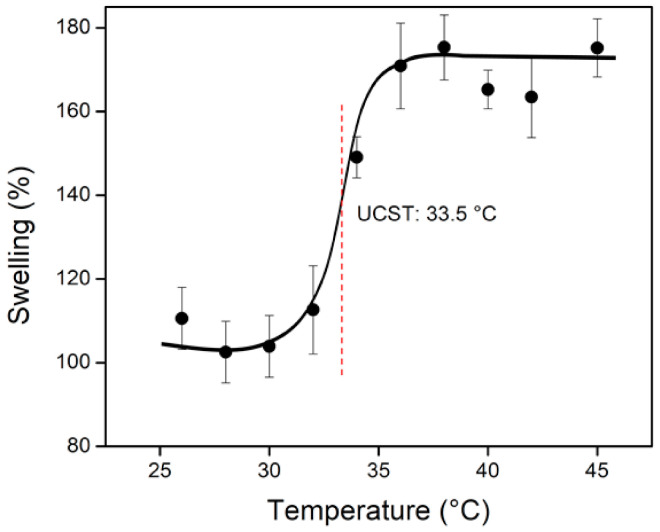
USCT of the binary grafted system (*net*-CS)-*g*-NVCL/AAc (32% graft).

It is known that (*net*-CS)-*g*-NVCL presents a LCST around 30–32 °C, depending on the grafting percentage [7]. In other words, polymers chains are expanded below this temperature and in a collapsed state above this temperature. In contrast, (*net*-CS)-*g*-NVCL/AAc presented the opposite behavior, in which grafted chains were collapsed when the temperature was below the critical temperature. By comparison, the expanded state predominated when the temperature was above this point. Thus, the grafted system showed an upper critical solution temperature (UCST) at 33.5 °C (Figure 9). These data were consistent with the results reported by other research teams [35,36,37], in which copolymers of NVCL showed an UCST rather than a LCST. This behavior was driven by the combined effect of hydrogen bonding between the carboxylic acid groups and hydrophobic interaction among the caprolactam moieties [38]. Amine groups of *net*-CS and carboxylic acid groups of AAc can form small aggregates by electrostatic interaction and hydrogen bonding. When the temperature increased, these interactions were broken and the repulsion forces between the polymeric chains predominated, generating the swollen or expanded state.

#### 3.3.6. Determination of the Critical pH of the Systems

**Figure 10:**
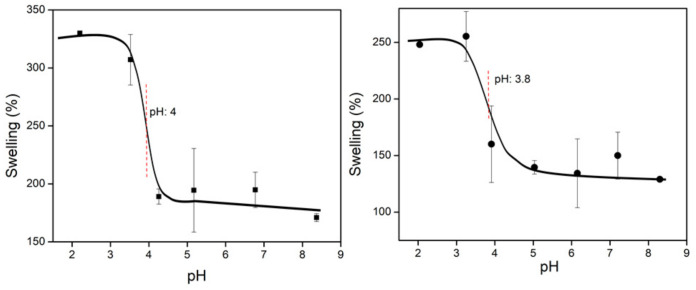
Critical pH at room temperature of *net*-CS (■) (**left**), and (*net*-CS)-*g*-NVCL/AAc (32% graft) (●) (**right**).

As observed in Figure 10, the critical pH values of *net*-CS and (*net*-CS)-*g*-NVCL/AAc differed slightly, obtaining values of 4.0 and 3.8, respectively. This point is usually close to the pKa of the monomer/polymer, but any change by the addition of another monomer or crosslinking changes this value. The pKa of CS has been reported to be between 6.17 and 6.51, depending in the molecular weight and degree of deacetylation [39]. However, this value can change during the crosslinking process because secondary amines are formed. Thus, the grafted material showed a slightly lower critical pH due to hydrogen bond interactions between the amine groups of *net*-CS and the carboxylic groups of the grafted chains. Although the swelling percentage was different in the two systems, this confirmed that the grafted system is more hydrophobic due to the insertion of NVCL chains.

### 3.4. Drug Loading and Release

It is important to note that pristine *net*-CS and (*net*-CS)*-g*-NVCL/AAc) were tested to release diclofenac and vancomycin; however, the latter could not be loaded due to the large volume of this molecule. This hindered the molecules’ diffusion in the pores of the grafted binary system, because the molecules were too large to allow them to diffuse effectively into the polymer matrix. 

**Figure 11:**
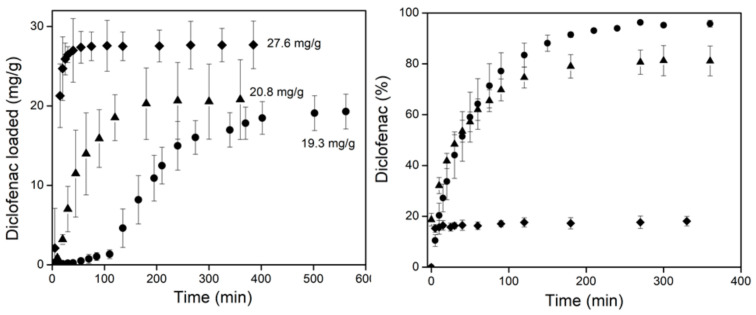
Loading (**left**) and release (**right**) profiles of diclofenac for different systems. (▲) *net*-CS and (●) (*net*-CS)-*g*-NVCL/AAc (32% graft) at pH 6.5 and 40 °C; (◆) (*net*-CS)-*g*-NVCL/AAc (32% graft) preactivated at pH 5 and 40 °C.

The grafted samples were swollen in water or a buffer solution (pH 5) to protonate the amine groups, allowing us to determine if the positive charge (preactivated sample) can improve the loading of diclofenac. Figure 11 shows the behavior of both *net*-CS and (*net*-CS)-*g*-NVCL/AAc in the different experimental conditions.

The samples loaded similar amounts of diclofenac at pH 6.5, but with differing behavior. Although the binary grafted sample released about 95% of the loaded drug in 200 min, the crosslinked CS only released about 80% in the same period. Because the grafted system has a UCST of 33 °C, it allowed a better release since the grafted chains were able to expand at a physiological temperature. Similarly, the apparent mechanical properties were improved in the swollen state, enhancing their handling. 

It was also noted that, when the grafted *net*-CS was first swollen in a pH of 5, the loading was higher (27.6 mg g^−1^). However, the release percentage was less than 20%. This is because diclofenac is a salt that contains a negative charge that adhered to the protonated CS by electrostatic interactions. These results suggest that the grafted system may be used in biomedical applications in which a material with antimicrobial properties is needed, because it can be loaded with a negatively charged antibacterial that will be retained in the material and protect it against microbial attacks.

Although CS networks can be used alone as a drug delivery system, the release time and the kind and quantity of the drug can be regulated through modifications such as graft copolymerization [40]. In this case, (*net*-CS)-*g*-NVCL/AAc showed an UCST, which is uncommon, but helped to release more diclofenac. This change in its response was caused by the random binary grafting. Previously, NVCL was grafted separately and did not exhibit this behavior [41,42]. Other systems reported in the literature showed that binary grafting was useful for enhancing different CS properties depending on the grafted monomers. For example, Sharma et al. (2014) grafted glycidyl methacrylate (GMA) onto chitosan using different comonomers (acrylic acid, acrylonitrile and acrylamide), obtaining copolymers that were capable of loading up to seven-fold more diclofenac than chitosan. Moreover, the copolymers showed that the release could be controlled by the pH of the medium [43]. Ortiz et al. (2018) prepared hydrogel films of maleoyagarose-*g*-PNIPAAm and chitosan, which presented better thermal, swelling, and release properties. In the obtained result, 53% of diclofenac was released after 24 h at 37 °C and pH 6 [44].

## 4. Conclusions

The synthesis of a dual-responsive system based on *net*-CS was achieved by grafting NVCL and AAc using the gamma radiation technique. The optimum condition to obtain the (*net*-CS)-*g*-NVCL/AAc system was the direct method, with a maximum grafting percentage of about 44% at 10 kGy. FTIR confirmed the grafting reaction and SEM showed the presence of interconnected pores in the structure, which is an important feature for a drug delivery system. Despite NVCL having a LCST around 30–32 °C, the (*net*-CS)-*g*- NVCL/AAc system showed an UCST at 33.5 °C. This occurred because of the different interactions taking place in the polymeric chains, mainly of hydrogen bonds and electrostatic interactions between carboxylic acid groups (–COOH) of AAc and amine groups (-NH) of CS. This change in the thermoresponsive behavior was previously observed in different NVCL copolymers by other research teams, who also reported an UCST in systems grafted with NVCL. The critical pH value of the binary grafting was observed to be 3.8, which was slightly lower than that of *net*-CS due to the introduction of the carboxylic acid groups. Moreover, the swelling percentage of the grafted system was lower, which improved the apparent mechanical properties of the samples, and allowed them to be easily handled without breaking up. Preliminary studies of drug retention were not entirely successful because the sizes of the pores were too small for the diffusion of molecules as large as those of vancomycin. However, diclofenac had better retention because of its small size and negative charge. The load and release of diclofenac showed that the grafted system (32%) was able to load 19.3 mg g^−1^ and release about 95% at 200 min, in comparison to crosslinked chitosan, which only released 80% in the same period. This was possibly caused by the UCST, which allows the pores of the grafted system to be open at the physiological temperature (37 °C). Moreover, when the grafted sample was protonated, the loaded drug was higher (27 mg g^−1^), but only 20% was released because of the strong electrostatic interactions between the sample and diclofenac.

Carrying out more studies with different drugs will be necessary to identify a successful means for their retention and release. 

## Data Availability

The data presented in this study are available on request from the corresponding author.
